# Supplementary material for: A whole-animal phenotypic drug screen identifies suppressors of atherogenic lipoproteins
Source: eLife. 2026 Aug 27;14:RP105314. doi: 10.7554/eLife.105314 (PMC13521566; doi:10.7554/eLife.105314)
Supplement: Supplementary file 2 [file elife-105314-code1.zip › JHDL_primary_result/Verteporfin/Verteporfin_JHDL_34_021_026_2020_01_31_11_11.pdf]

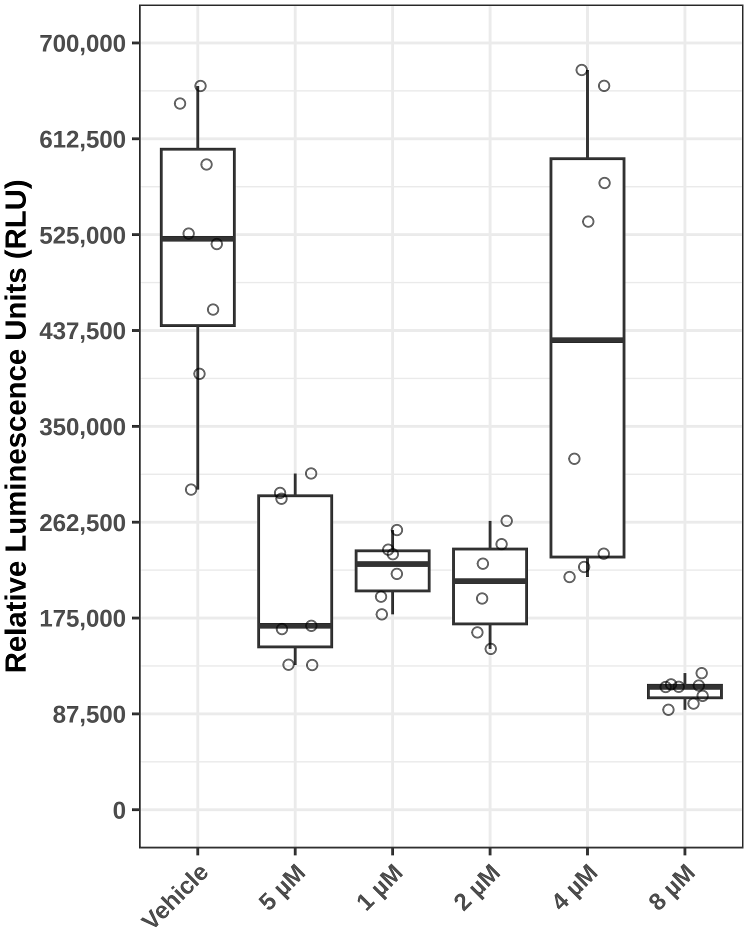
Relative Luminescence Units (RLU)
700,000
612,500
525,000
437,500
350,000
262,500
175,000
87,500
0
Vehicle
5 μM
1 μM
2 μM
4 μM
8 μM
